# The P3N-PIPO Protein Encoded by Wheat Yellow Mosaic Virus Is a Pathogenicity Determinant and Promotes Its Pathogenicity through Interaction with NbRLK6 in *Nicotiana benthamiana*

**DOI:** 10.3390/v14102171

**Published:** 2022-09-30

**Authors:** Runpu Miao, Zhuangxin Ye, Stuart MacFarlane, Yanjun Li, Qianzhuo Mao, Yanzhen Tian, Zhiping Deng, Zongtao Sun, Jian Yang, Junmin Li, Fei Yan, Jianping Chen, Chulang Yu

**Affiliations:** 1 State Key Laboratory for Managing Biotic and Chemical Threats to the Quality and Safety of Agro-Products, Key Laboratory of Biotechnology in Plant Protection of Ministry of Agriculture and Zhejiang Province, Institute of Plant Virology, Ningbo University, Ningbo 315211, China; 2 Cell and Molecular Sciences, James Hutton Institute, Invergowrie, Dundee DD2 5DA, UK; 3 State Key Laboratory for Managing Biotic and Chemical Threats to the Quality and Safety of Agro-Products, Institute of Virology and Biotechnology, Zhejiang Academy of Agricultural Sciences, Hangzhou 310021, China

**Keywords:** WYMV, P3N-PIPO, pathogenicity, NbRLK6

## Abstract

Similarly to other potyvirids, the bymovirus wheat yellow mosaic virus (WYMV) encodes a P3N-PIPO protein that is expressed by frameshifting occurring within the open reading frame of the P3 protein. P3N-PIPO is known to be essential for the cell-to-cell movement of several potyviruses, but this has not yet been confirmed for the WYMV. Here, we show that the WYMV P3N-PIPO protein influences disease symptom formation. Infection of *Nicotiana benthamiana* plants with a potato virus X (PVX)-based vector carrying the WYMV P3N-PIPO gene induced more severe disease symptoms and resulted in higher virus accumulation levels than did infection with PVX lacking the P3N-PIPO gene. *N. benthamiana* P3N-PIPO-interacting proteins were identified through co-immunoprecipitation (Co-IP) coupled with LC-MS/MS (mass spectrometry), and the interaction between P3N-PIPO and the *N. benthamiana* receptor-like kinase NbRLK6 was further verified by Co-IP and bimolecular fluorescence complementation (BiFC) of transiently-expressed proteins. Furthermore, our investigation showed that the disease symptom severity and accumulation level of PVX-P3N-PIPO were decreased in *N. benthamiana* plants when *NbRLK6* expression was reduced by tobacco rattle virus-induced gene silencing.

## 1. Introduction

Wheat yellow mosaic virus (WYMV), belonging to the genus *Bymovirus* and being transmitted by the plasmodiophorid *Polymyxa graminis*, can cause the soilborne viral disease wheat yellow mosaic (WYM) [1]. WYM is one of the most serious diseases affecting wheat production in East Asia [2].

Previous examination of the WYMV genome suggested that it produces ten proteins (P3, 7k, CI, 14k, VPg, NIa, NIb, CP, P1 and P2) derived by proteolytic cleavage of the two polyproteins encoded by the two viral RNAs [1]. In recent years, a small overlapping coding sequence, which is termed PIPO (pretty interesting potyviridae ORF), was discovered by bioinformatics analysis within the genome sequences of viruses in the Potyviridae family [3]. A novel protein named P3N-PIPO, with the PIPO segment being encoded in a different frame within the P3 cistron, is expressed by a viral polymerase slippage mechanism [4,5,6]. In previous studies, P3N-PIPO was shown to be required for efficient cell-to-cell movement of several potyviruses [7,8,9,10,11]. Here we report the results of experiments expressing the WYMV P3-PIPO protein from potato virus X (PVX). This study shows that the WYMV P3NPIPO protein is a pathogenicity determinant, which enhances the pathogenicity of PVX in *N. benthamiana*. To better understand the mechanism by which WYMV P3N-PIPO influences disease symptom development, we identified host plant proteins that physically interact with P3N-PIPO. Interaction with one of these, NbRLK6, a member of the receptor-like kinase (RLKs) family, was confirmed by co-immunoprecipitation and BiFC assays. Furthermore, our investigation showed that silencing of *NbRLK6* expression in *N. benthamiana* decreased both the severity of PVX disease symptom production and virus accumulation. Previous Co-IP studies of other viruses have not identified RLK6 as a P3-PIPO binding partner. This is the first report of RLK6 as a binding partner of P3-PIPO from any virus.

## 2. Materials and Methods

### 2.1. Plasmid Construction

For the pathogenicity assessment of P3N-PIPO and Co-IP/MS and BiFC assays, the constructs were generated using Gateway technology (Invitrogen Life Technologies, Carlsbad, CA, USA). The full-length sequences of WYMV P3N-PIPO (contain G_2_A_7_ motif) and P3N-PIPO-AS (antisense) were amplified with the corresponding primer pairs (Table S1), from the constructed vector PVX-WYMV P3N-PIPO(FS-1)-GFP in our previous study [6]. The NbRLK6 gene was amplified with the corresponding primer pair (Table S1), from *N. benthamiana* cDNA. The PCR fragments of P3N-PIPO, P3N-PIPO-AS and NbRLK6 were cloned into the pDONR207 entry vector through Gateway technology (Invitrogen Life Technologies, Carlsbad, CA, USA). Then, we subcloned into the destination vectors PVX-attB (Figure S1), pcL112-nYFP, pcL113-cYFP (these destination vectors were kind gifts from Dr. Stuart MacFarlane) and pBA-Flag-4Myc-DC (kept by Dr. Yanjun Li), via the Gateway^®^ LR Recombination reaction (Invitrogen Life Technologies, Carlsbad, CA, USA) following the manufacturer’s instructions. PVX (pGR106) was a kind gift from David Baulcombe’s laboratory, PVX-attB was from Dr. Sean Chapman’s laboratory and PVX-P19 was from Dr. Stuart MacFarlane’s laboratory.

In Co-IP assays, the constructs were generated through ligation-independent cloning (LIC). P3N-PIPO and NbRLK6 were amplified by PCR and were later cloned into pCV-3×myc-N1 and pCV-3×flag-N1, respectively. 

To silence NbRLK6 expression in *N. benthamiana*, a 200 bp fragment of the *NbRLK6* sequence was amplified and positive-sense-inserted into the pTRV2-LIC vector (kindly provided by Professor Yule Liu from Tsinghua University, Beijing, China) through the LIC method to generate pTRV2-NbRLK6. 

### 2.2. Plant Growth and Agrobacterium Infection of Plants 

*N. benthamiana* were grown in a glasshouse under 25 °C—22 °C ± 2 °C (day: night)—and 16 h/8 h (light/dark) photoperiod conditions. 

Agrobacterium cultures containing the experimental plasmids were infiltrated into the leaves of 4-leaf-stage *N. benthamiana* plants using the previously described method [12].

### 2.3. Co-IP/MS

Agrobacterium cultures carrying the plasmid pBA-Flag-4Myc-P3N-PIPO infiltrated *N. benthamiana leaves*, and the systemic leaves were collected for the total protein extraction. The P3N-PIPO complexed with interacting plant proteins was isolated following the method described previously [13].

Then, the washed beads carrying P3N-PIPO and co-precipitated host plant proteins were sent for MS analysis using a quadrupole-orbitrap mass spectrometer, Q Exactive HF (Thermo Scientific, Waltham, MA, USA). Finally, data were analyzed according to the previous description [14,15]. 

### 2.4. Bioinformatics Analysis

For functional annotation and classification, all co-precipitated plant proteins were aligned against the InterProScan database (https://www.ebi.ac.uk/interpro/about/interproscan/, accessed on 4 August 2022). Gene Ontology (GO, http://www.geneontology.org, accessed on 6 August 2022) and Kyoto Encyclopedia of Genes and Genomes (KEGG, http://www.genome.jp/kegg/, accessed on 6 August 2022) analyses were performed using InterProScan and KOBAS (http://kobas.cbi.pku.edu.cn/, accessed on 15 August 2022), respectively. Additionally, enrichment analysis of the P3N-PIPO-interacting proteins was completed using the R package clusterprofile.

### 2.5. BiFC Assay 

Plasmids pcL-cYFP-P3N-PIPO and pcL-nYFP-NbRLK6 were separately transformed into *A. tumefaciens* strain GV3101. The two agrobacterium cultures were mixed at a 1:1 (*v*/*v*) ratio and infiltrated into the leaves of *N. benthamiana* plants. Mixed agrobacterium cultures carrying pcL-cYFP-P3N-PIPO and pcL-nYFP-GUS served as a non-interacting control. At 48 h post-infiltration (hpi), the leaves were collected and examined under a Leica TCS SP8X confocal microscope (Leica Microsystems, Bannockburn, IL, USA).

### 2.6. Co-IP Transient Assay 

For transient Co-IP assays, mixed agrobacterium cultures carrying the plasmids pCV-3×myc-P3N-PIPO and pCV-3×flag-NbRLK6 or pCV-3×myc-P3N-PIPO and pCV-3×flag-GFP were infiltrated into *N. benthamiana* leaves. At 2dpi, total proteins were extracted from 0.4 g of powdered leaf using 1.2 mL IP buffer (40 mM Tris-HCl, pH 8.0, 100 mM NaCl, 1 mM EDTA, 1 mM DTT, 0.2% Triton X-100, 1 mM PMSF, 1% glycerol, 1 pellet/12.5 mL) and complete EDTA-free protease inhibitor (Roche Diagnostics, Basel, Switzerland). The Co-IP was performed as described previously [16].

### 2.7. Virus-Induced Gene Silencing in N. benthamiana 

Plasmids pTRV1, pTRV2 and pTRV2-NbRLK6 (Table S2) were individually transformed into *A. tumefaciens* strain GV3101. Thereafter, the mixed agrobacterium cultures of pTRV1 and TRV:NbRLK6, were infiltrated into the leaves of 3-leaf-stage *N. benthamiana* plants. The combination of TRV:00 (pTRV1 and pTRV2) was used as a negative control for the VIGS experiments. More details of the VIGS methodology were described previously [17].

### 2.8. Quantitative Reverse Transcription Polymerase Chain Reaction (qRT-PCR) 

Total RNA was extracted from leaf tissue using TRIzol reagent (Invitrogen Life Technologies, Carlsbad, CA, USA) following the manufacturer’s protocols. The complementary DNA (cDNA) was synthesized using the HiScript III RT SuperMix for qPCR (plus gDNA wiper) (Vazyme, Nanjing, China). qRT PCR was performed on the LightCycler 480^®^ Real-Time PCR System (Roche Diagnostics, Basel, Switzerland) using the Universal Blue qPCR SYBR Master Mix (Yeasen, Shanghai, China). The NbUBC (Ubiquitin C) gene was used as an internal reference in the assays. The results were analyzed by the 2-ΔΔCT method [18].

### 2.9. Western Blot (WB) Analysis 

Total proteins were extracted from 1 g of the infiltrated *N. benthamiana* leaves in 20% SDS lysis buffer (100 mM Tris-HCl (pH6.8), 20% SDS, 2% β-mercaptoethanol), separated through 12% SDS-PAGE and transferred onto a PVDF membrane. The membrane was then incubated with PVX CP rabbit antibody (1:8000, HuaBio, Hangzhou, China) or commercial protein tag antibodies (1:5000, TransGen, Beijing, China), and later with the IgG-HRP secondary antibody (1:10,000, TransGen, Beijing, China). Finally, the blot was treated with the ECL solution (Sangon, Shanghai, China) and examined using a GE Amersham imager 680.

## 3. Results

### 3.1. P3N-PIPO Enhanced the Pathogenicity of PVX in N. benthamiana

**Figure 1:**
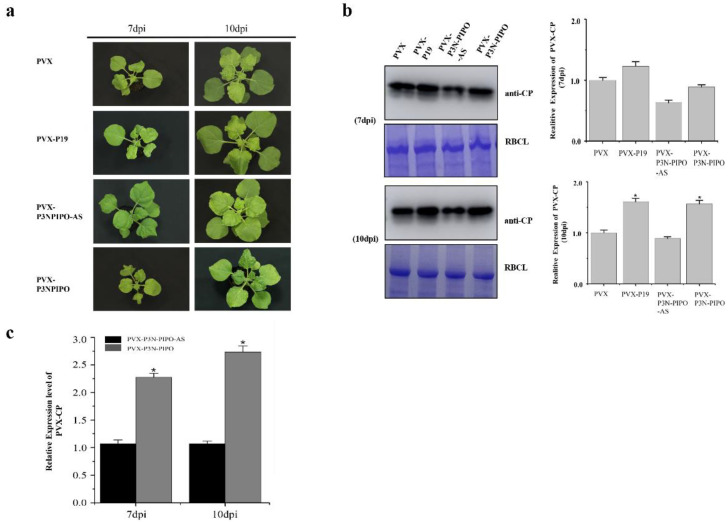
WYMV P3N-PIPO can affect PVX pathogenicity in *N. benthamiana*. (**a**) Disease symptom map of *N. benthamiana* plants coinfected with PVX, PVX-P3N-PIPO, PVX- P3N-PIPO-AS and PVX-P19. (**b**) Immunoblotting analysis of PVX CP accumulation in systemically infected leaves. Coomassie staining of the rubisco large subunit (RBCL) was performed to makes a loading control. The bands were quantified as shown on the right. (**c**) qRT-PCR of PVX CP RNA as a measure of PVX infection level in systemic leaves. Error bar indicate standard error from three individual experiments. Samples from twenty plants were pooled for analysis in each experiment. Results are represented by the mean ± standard deviation (SD) of three independent experiments. * indicates significant difference between control plants (PVX-P3N-PIPO-AS) and PVX-P3N-PIPO-infected plants at *p* ≤ 0.05.

To investigate the properties of the WYMV P3N-PIPO protein, the P3N-PIPO gene was inserted into a PVX-based vector (pGR106), enabling its expression in *N. benthamiana* plants [19,20]. In addition, either the P19 gene of tomato bushy stunt virus (TBSV), a well-studied viral pathogenicity determinant, or an antisense version of the WYMV P3-PIPO gene (P3N-PIPO-AS), was cloned into pGR106 as an experimental control. According to our results, the PVX-P3N-PIPO-inoculated plants exhibited irregular leaf mottling at 7dpi, and then severe irregular leaf mottling and leaf deformation at 10 dpi; PVX-P3N-PIPO-AS-inoculated plants showed only very mild mosaic symptoms at 10 dpi (Figure 1a). The top leaves of PVX-EV (empty vector)-inoculated plants exhibited shrinking at 7dpi; then the leaves began unfurling and exhibited chlorisis at 10dpi (Figure 1a). The symptoms of the PVX-P19-infected plants were more severe than PVX-EV; the top leaves exhibited more curling. The symptoms of the PVX-P3N-PIPO-infected plants were different from those of PVX-P19-infected plants. The main symptom of PVX-P19-infected plants was severe leaf curling. PVX-P3N-PIPO-infected plants exhibited chlorosis, severe irregular leaf mottling and leaf deformation (Figure 1a). 

To further investigate the effect of P3N-PIPO expression on PVX infection, the systemically infected leaves from PVX-EV-, PVX-P19-, PVX-P3N-PIPO-AS- and PVX-P3N-PIPO-inoculated plants were analyzed by Western blotting and qRT-PCR. The results showed that there were higher levels of PVX coat protein (CP) and viral RNA in the PVX-P3N-PIPO-inoculated plants than in the PVX-P3N-PIPO-AS plants (Figure 1b, c). Consequently, it was concluded that the WYMV P3N-PIPO protein could act a determinant of PVX pathogenicity in *N. benthamiana* plants.

### 3.2. Identification and Analysis of Plant Proteins Interacting with WYMV P3N-PIPO 

Investigation of protein–protein interactions is an essential process to understanding the underlying processes taking place during virus–host interactions. To identify potential interacting host factors, the WYMV P3N-PIPO tagged with both FLAG and Myc epitopes was transiently expressed in *N. benthamiana* leaves by agroinfiltration. Potential complexes of P3N-PIPO and host proteins were initially immunoprecipitated using the FLAG tag, and then, after further washing steps, were re-precipitated using the Myc tag. This approach greatly reduces the co-precipitation of non-specific binding proteins. Subsequently, the remaining captured proteins were identified using LC-MS/MS. 

This analysis identified 117 potential P3N-PIPO-interacting proteins (File S1). To understand in more detail the putative functional processes associated with the candidate P3N-PIPO-interacting proteins, GO analysis was conducted to provide known information about the biological processes (BP), cellular components (CC) and molecular functions (MF) of the candidate proteins; and KEGG pathway analysis was performed to show other interactions known for the candidate P3N-PIPO-binding proteins. 

**Figure 2:**
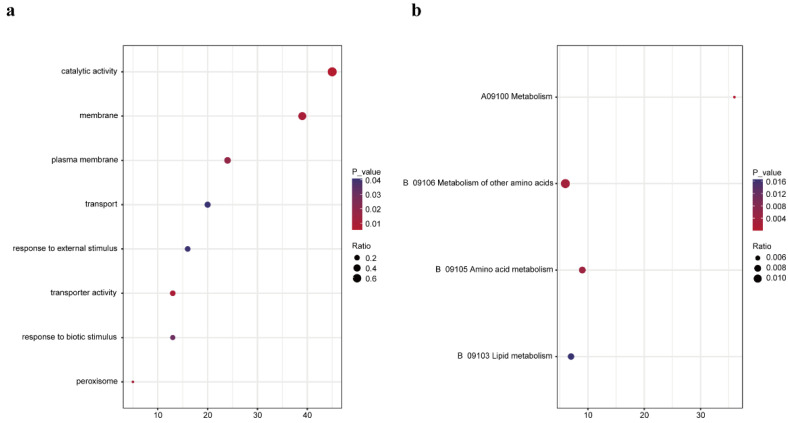
(**a**) GO annotation of candidate interacting proteins in three categories: biological processes (BP), cellular components (CC) and molecular functions (MF). The size of a dot indicates the ratio of candidate interacting proteins in the GO item, and the color of the dot represents the *p* value of the GO item. (**b**) KEGG pathway enrichment analysis of candidate interacting proteins with the top 10 pathways obtained by KEGG enrichment analysis. The size of a dot indicates the ratio of candidate interacting proteins in the pathway, and the color of the dot represents the *p* value of the pathway.

According to our results, the enriched GO-CC terms were mainly peroxisome, plasma membrane and membrane; the enriched MF terms included transporter activity and catalytic activity; and the BP terms included lipid metabolic process and catabolic process (Figure 2a). The top 10 KEGG pathways enriched by the target genes included amino acid metabolism, lipid metabolism, metabolism of other amino acids and metabolism pathways (Figure 2b).

### 3.3. P3N-PIPO Interacts with NbRLK6

**Figure 3:**
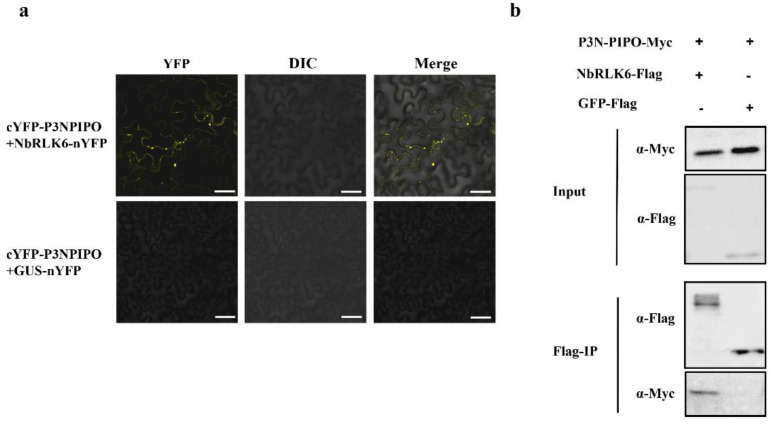
Verification of the interaction between the WYMV P3N-PIPO protein and NbRLK6 in agro-infiltrated *N. benthamiana.* (**a**) Detection of the interaction between P3N-PIPO and NbRLK6 by bimolecular fluorescence complementation test. Fluorescence, bright field (DIC) and merged images of the lower epidermises of leaves infiltrated with cYFP- and nYFP-fusion constructs were taken using a laser scanning confocal microscope. Bar = 50 μm. (**b**) Detection of the interaction between P3N-PIPO and NbRLK6 by Co-IP assay. Protein extracts were precipitated using an anti-FLAG antibody. Input refers to the total protein extract. Flag-IP refers to immunoprecipitated proteins, which were detected using either anti-FLAG or anti-Myc antibodies. “+” and “−” represent the presence or absence of specific constructs in the agroinfiltration mixture.

Among the 117 potential P3N-PIPO-interacting proteins identified in our screen, we selected a putative receptor-like kinase NbRLK6 for further study, as RLK proteins are known to play a prominent role in various plant–pathogen interactions [21]. Biomolecular fluorescence complementation (BiFC) assays were performed to verify the interaction of WYMV P3N-PIPO and NbRLK6 in plants. Thus, using agroinfiltration, we transiently co-expressed cYFP-P3N-PIPO and nYFP-NbRLK6 in *N. benthamiana* leaves. This resulted in the production of a YFP fluorescence signal in the cell cytoplasm at 48 h post-infiltration, suggesting physical interaction between these two proteins. However, no YFP fluorescence was observed in leaves co-infiltrated with cYFP-P3N-PIPO and the non-interacting protein nYFP-GUS, ruling out the possibility of YFP auto-fluorescence from the cYFP-P3N-PIPO protein (Figure 3a).

As a further test for interaction, WYMV P3N-IPO tagged with Myc and NbRLK6 tagged with FLAG were co-expressed by agroinfiltration in *N. benthamiana* leaves. As a non-interaction control, WYMV P3N-PIPO-Myc was co-expressed with GFP-FLAG. Leaf extracts were precipitated using a bead-immobilized anti-FLAG antibody, and the collected proteins were examined by Western blotting. In these experiments, P3N-PIPO was co-precipitated in combination with NbRLK6-FLAG, but not with GFP-FLAG (Figure 3b).

**Figure 4:**
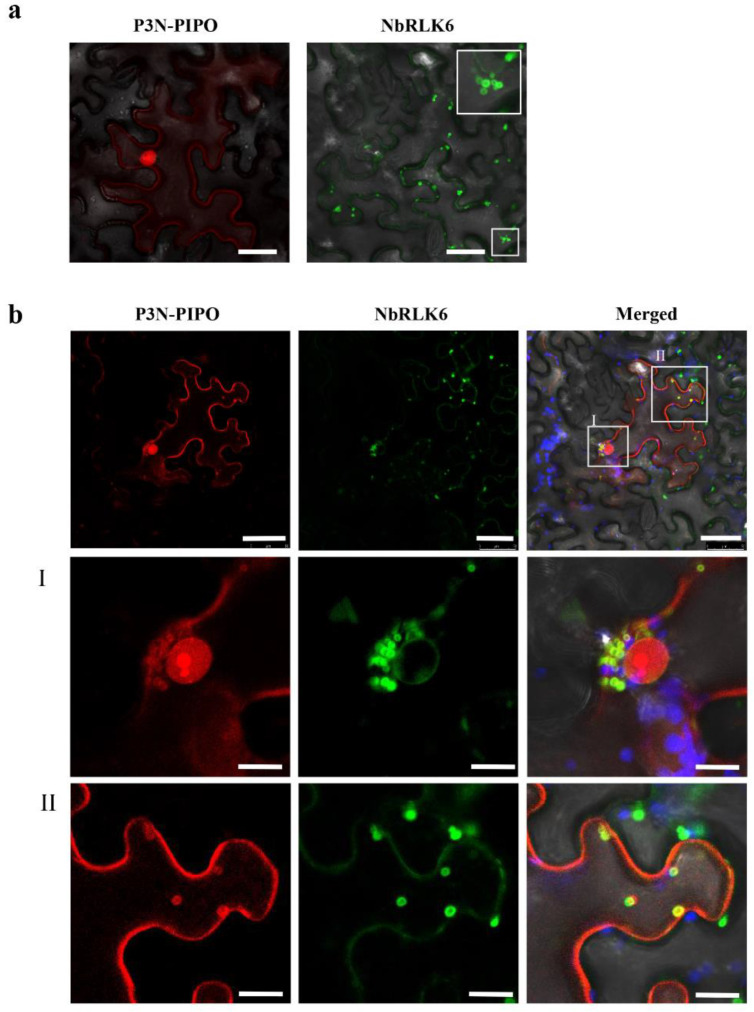
Subcellular localization of NbRLK6 and its colocalization with WYMV P3N-PIPO. (**a**) Subcellular localization of P3N-PIPO-mRFP and NbRLK6-GFP in *N. benthamiana* plants examined by confocal microscopy at 48 hpi. Scale bar, 10 μm. (**b**) **I**. Scale bar, 25 μm. **II**. Scale bar, 50 μm.

To study whether NbRLK6 and P3N-PIPO co-localized in plants, leaves were infiltrated with constructs expressing NbRLK6 fused to the green fluorescent protein (NbRLK6-GFP) and P3N-PIPO fused to the red fluorescent protein (P3N-PIPO-mRFP). When expressed alone, P3N-PIPO (mRFP) was associated with the nucleus and cytomembrane, and NbRLK6 (GFP) aggregated to form vesicles (Figure 4a). While the relatively weak fluorescence precluded visualization of P3N-PIPO-RFP at the plasmodesmata (PD), fusion of P3N-PIPO to GFP, as expected, clearly showed localization of P3N-PIPO with PD (Figure S2). The co-expression led to the redistribution of P3N-PIPO to the vesicles of NbRLK6 (Figure 4b), which further verified the interaction between P3N-PIPO and NbRLK6, as identified using Co-IP (Figure 3).

### 3.4. Silencing of NbRLK6 Expression in N. benthamiana Decreases PVX Accumulation

**Figure 5:**
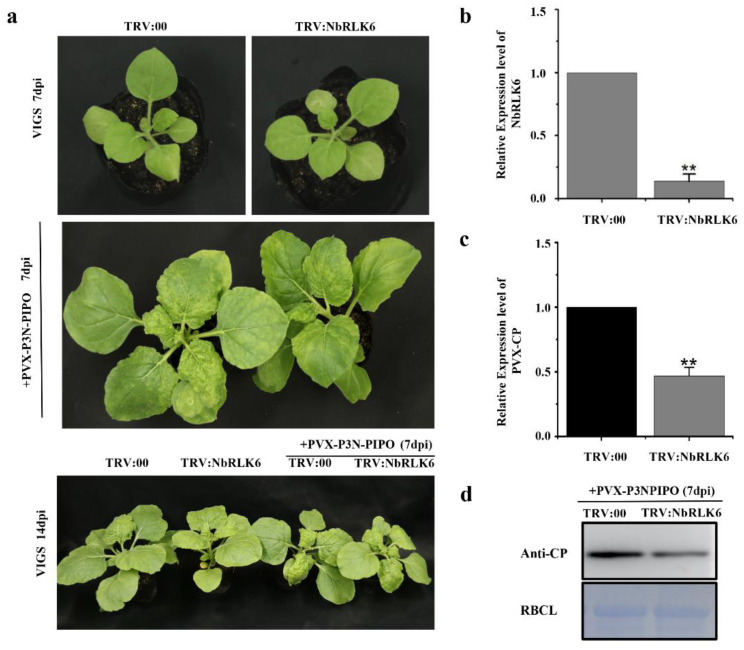
Silencing of NbRLK6 mitigated the pathogenicity of PVX-P3N-PIPO in *N. benthamiana*. (**a**) Strong symptoms of leaf chlorosis and malformation caused by PVX infection in control plants (TRV:00) and more mild PVX symptoms in NbRLK6-silenced plants (TRV: NbRLK6) were observed at 14 dpi. (**b**) qRT-PCR analysis of NbRLK6 transcript levels in TRV:00 and TRV:NbRLK6-infected *N. benthamiana* plants (7 dpi, before superinfection by PVX). NbUBC served as an internal control. Bars indicate standard error from three individual experiments. (**c**) qRT-PCR assay of relative levels of PVX CP RNA in non-silenced and NbRLK6-silenced plants at 14 days post TRV infection. Bars indicate standard error from three individual experiments. Twenty plants were sampled and pooled together in each experiment. (**d**) Immunoblotting analysis of the accumulation of PVX CP in control and NbRLK6-silenced plants 14 days post TRV infection. Asterisks indicate significant differences by Student’s *t*-test compared with the control (**, *p* < 0.01).

To determine the role of NbRLK6 in virus infection, *NbRLK6* expression was silenced in *N. benthamiana* using the TRV-based VIGS vector and inoculated with empty vector (TRV:00) as a control. By 7 dpi, the TRV:00 and TRV:NbRLK6-inoculated plants showed no obvious symptoms (Figure 5a); however, the qRT-PCR assay suggested that the mean transcription level of *NbRLK6* in TRV:NbRLK6-inoculated plants was reduced by approximately 80% compared with that in TRV:00-inoculated control plants (Figure 5b). Thereafter, the effects of NbRLK6 on PVX-P3N-PIPO accumulation and symptom induction were investigated. By 14 dpi, the TRV:00 control plants inoculated with PVX-P3N-PIPO exhibited severe leaf curling and mosaic symptoms, whereas only mild leaf curling symptoms were observed on the NbRLK6-silenced plants after inoculation with PVX-P3N-PIPO (Figure 5a). qRT-PCR and Western blotting results showed that, compared with control plants, the accumulation level of PVX was significantly reduced in the NbRLK6-silenced plants (Figure 5c,d). Taken together, these results indicate that NbRLK6 plays important roles in virus accumulation and the development of disease symptoms in PVX-infected plants.

## 4. Discussion

In plants, RLKs play a key role in regulating plant development and plant responses to environmental stimuli [22,23,24]. Many studies have revealed that RLKs are involved in plant defense against bacteria, fungi, oomycetes, and even insects [25]. 

A typical RLK contains an extracellular domain, a single-pass transmembrane domain, and a cytoplasmic kinase domain [26]. Usually, the extracellular domain of RLKs was involved in perceiving the invasion of pathogens. As viruses are intracellular pathogens, the involvement of RLKs in plant–virus interactions seems counterintuitive. However, more and more evidence shows that RLKs affect plant susceptibility to viruses, and in some cases, interact with viral proteins [27,28,29,30,31,32,33,34]. The nuclear shuttle protein (NSP) of bipartite geminiviruses acts as a virulence factor, which also binds to Arabidopsis RLKs, and thus inhibits their function in anti-viral defense [28,35,36,37]. Arabidopsis NSP-interacting kinases 1, 2, and 3 (AtNIK1-3) were the first RLKs identified to interact with viral protein [35]. The C4 protein of geminiviruses has been the most extensively studied in terms of the interaction between RLKs and viral proteins. Typically, the C4/AC4 protein of geminiviruses has been revealed to interact with RLKs in the CLAVATA 1 (CLV1) clade [25,38]. 

Numerous studies have indicated that P3N-PIPO is the movement protein of potyviruses [7,8,9,10,11]. In addition, P3N-PIPO determines the virulence of clover yellow vein virus (ClYVV) in both resistant and susceptible peas [39]. However, the mechanism underlying this phenomenon remains unresolved. Here, our study revealed that WYMV P3N-PIPO is a determinant of pathogenicity, which enhances the symptom severity and accumulation level of PVX in *N. benthamiana*. We discovered that NbRLK6, a member of the plant RLK family, interacts with WYMV P3N-PIPO, and confirmed the interaction by BiFC and Co-IP assays. Furthermore, reducing *NbRLK6* expression in *N. benthamiana* by TRV-VIGS decreased the accumulation of the chimeric virus PVX-P3N-PIPO and alleviated the viral disease symptoms. 

Intriguingly, over the past few years, most studies on the interaction between RLKs and viral proteins were focused on those of geminiviruses, which have DNA genomes and nuclear replication cycles [25]. Our study focused on the RNA virus PVX and suggests that more work should be performed on viruses from different families in order to more systematically study the interaction between RLKs and viral proteins. 

## Data Availability

The data presented in this study are available on request from the corresponding author.

## Supplementary Materials

The following supporting information can be downloaded at: https://www.mdpi.com/article/10.3390/v14102171/s1, Figure S1: Schematic representation of PVX-based vector; Figure S2: Subcellular localization of WYMV P3N-PIPO-GFP in *N.benthamiana* leaf cells; Table S1: List of primers used in this study; Table S2: The PCR amplified fragment for NbRLK6 VIGS experiment; File S1: List of *N. benthamiana* interactors of WYMV P3N-PIPO identified by Co-IP/MS.
