# Supplementary material for: The P3N-PIPO Protein Encoded by Wheat Yellow Mosaic Virus Is a Pathogenicity Determinant and Promotes Its Pathogenicity through Interaction with NbRLK6 in Nicotiana benthamiana
Source: Viruses. 2022 Sep 30;14(10):2171. doi: 10.3390/v14102171 (PMC9607425; doi:10.3390/v14102171)
Supplement: Supplementary file 1 [file viruses-14-02171-s001.zip › Table S1.pdf]

Table.S1. List of Primers Used in This Study

Primer Name	Sequence 5'-3'
Lic-NbRLK6-F:	CGACGACAAGACCGTCACCATGGCAGCCACTTTCCAGCGC
Lic-NbRLK6-R:	GAGGAGAAGAGCCGTCGAGCTTTTGCAGTAGCAATACT
NbRLK6-GW-F:	AAAAAGCAGGCTCCATGGCAGCCACTTTCCAGCGC
NbRLK6-GW-R:	AGAAAGCTGGGTCAGCTTTTGCAGTAGCAATACT
TRV-NbRLK6-F	CGACGACAAGACCGTCACCATGTGGCAACGAATTGGGGAAGA
TRV-NbRLK6-R	GAGGAGAAGAGCCGTCGGCCATTATAGGTCGTGGGCT
WYMV-P3NPIPO-F	TATGGAGCAGACAGCAGCCCAG
WYMV-P3NPIPO-R-(STOP)	TCTACGAAAGATAGGTATAGCGCGAA
Lic-P3NPIPO-F:	CGACGACAAGACCGTCACCATGGAGCAGACAGCAGCCCAG
Lic-P3NPIPO-R:	GAGGAGAAGAGCCGTCGCGAAAGATAGGTATAGCGCGAA
WYMV-P3NPIPO-GW-F	AAAAAGCAGGCTCCATGGAGCAGACAGCAGCCCAG
WYMV-P3NPIPO-GW-R	AGAAAGCTGGGTCCTACGAAAGATAGGTATAGCGCGAA
qRT-RLK6-F:	CGAATTAAGGATGCCCAAGA
qRT-RLK6-R：	TCTTTGCCGGTAAACAGCTT
qRT-ubc-F	TTTCGGTCCTGATGATACTCCC
qRT-ubc-R	CACAGAGCAAAGACTGGATTGA
qRT-PVXCP-F:	CCGCAGCTATCACTCCCAAA
qRT-PVXCP-F:	TGCAACAGTCGTTCCTGTGA
