# Supplementary material for: The P3N-PIPO Protein Encoded by Wheat Yellow Mosaic Virus Is a Pathogenicity Determinant and Promotes Its Pathogenicity through Interaction with NbRLK6 in Nicotiana benthamiana
Source: Viruses. 2022 Sep 30;14(10):2171. doi: 10.3390/v14102171 (PMC9607425; doi:10.3390/v14102171)
Supplement: Supplementary file 1 [file viruses-14-02171-s001.zip › Table S2.pdf]

The PCR amplified fragment for NbRLK6 VIGS

ATGTGCCCAGGATGTGACATGCGAAGATTCTGATGGTTGTGGCAGCGACCACACTGCACTTATAGCAGGTTT
GACATCAGGTCTGGGTGTGGCAGTGATTGCTGTTGTAATTGCAGTTTTTGTGTACAGACGTCATAAGCGAAT
TAAGGATGCCCAAGATCGACTAGCTCGCGAACGGGAAGATATTCTTAGCTCTGGGGGTGTAA
