# Supplementary figures and images for: The P3N-PIPO Protein Encoded by Wheat Yellow Mosaic Virus Is a Pathogenicity Determinant and Promotes Its Pathogenicity through Interaction with NbRLK6 in Nicotiana benthamiana
Source: Viruses. 2022 Sep 30;14(10):2171. doi: 10.3390/v14102171 (PMC9607425; doi:10.3390/v14102171)

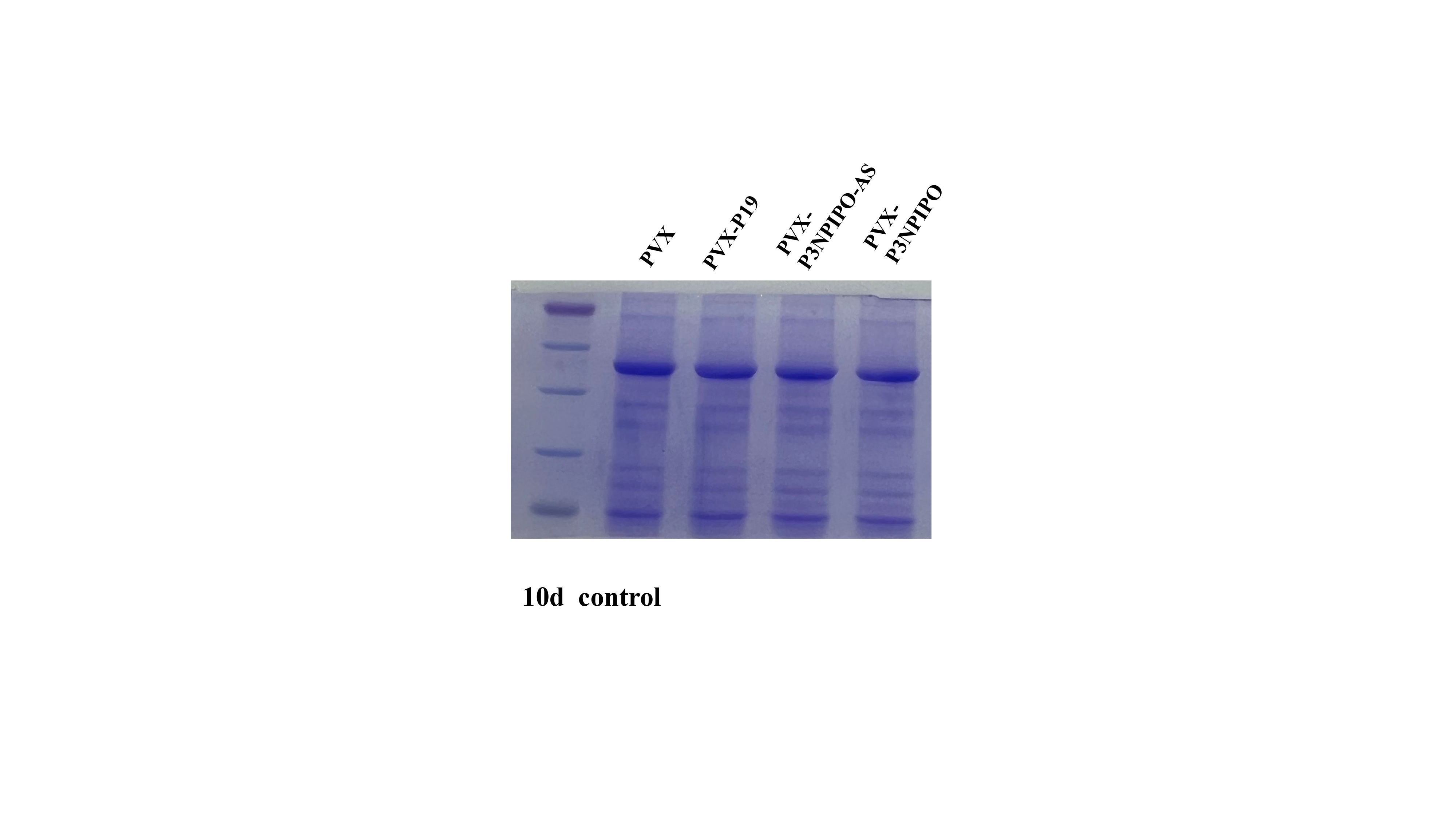

Supplement: Supplementary file 1 [file viruses-14-02171-s001.zip › Figs-original/Fig1-WB-original/Fig.1-10d-control1.tif]

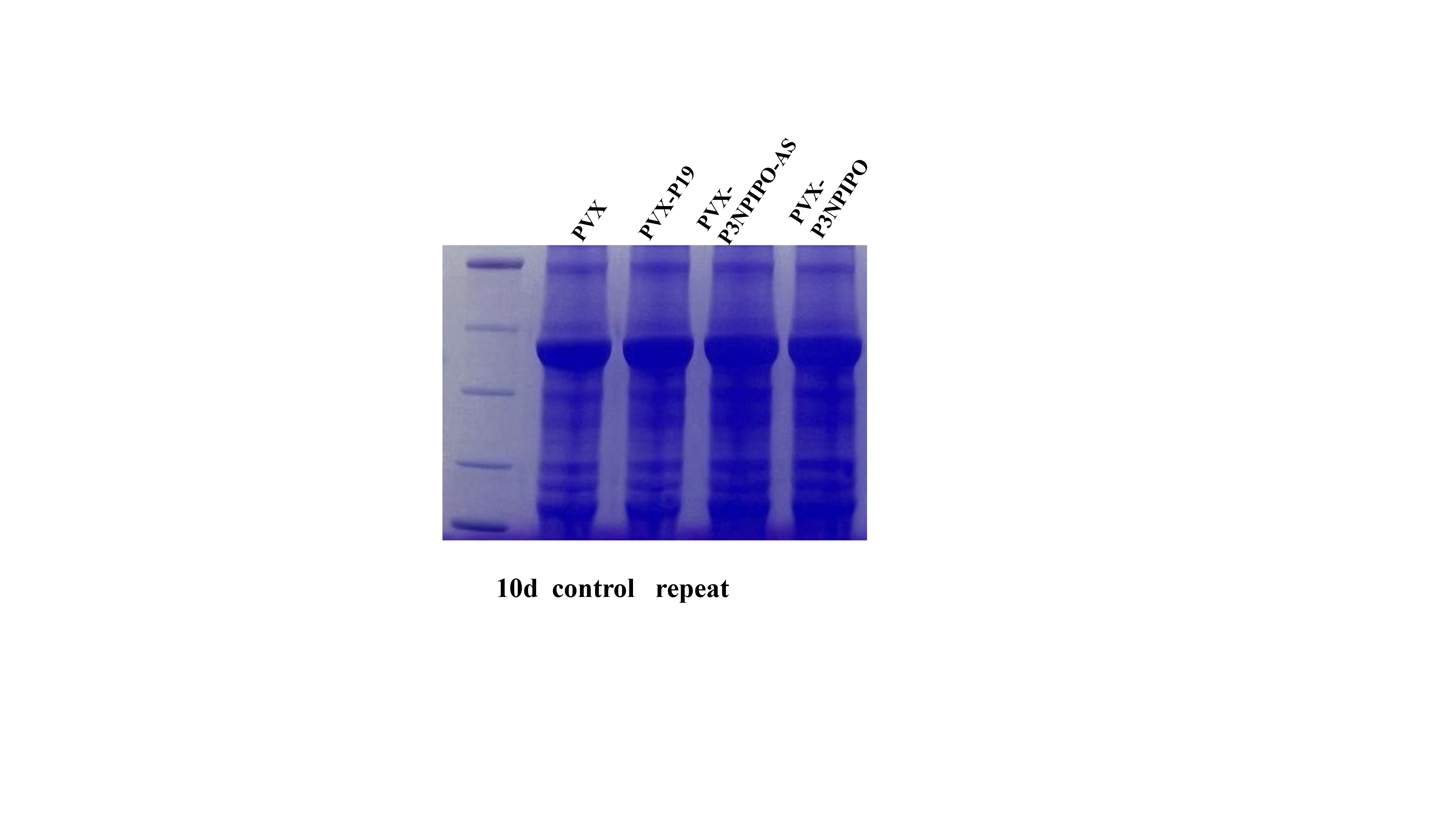

Supplement: Supplementary file 1 [file viruses-14-02171-s001.zip › Figs-original/Fig1-WB-original/Fig.1-10d-control2.tif]

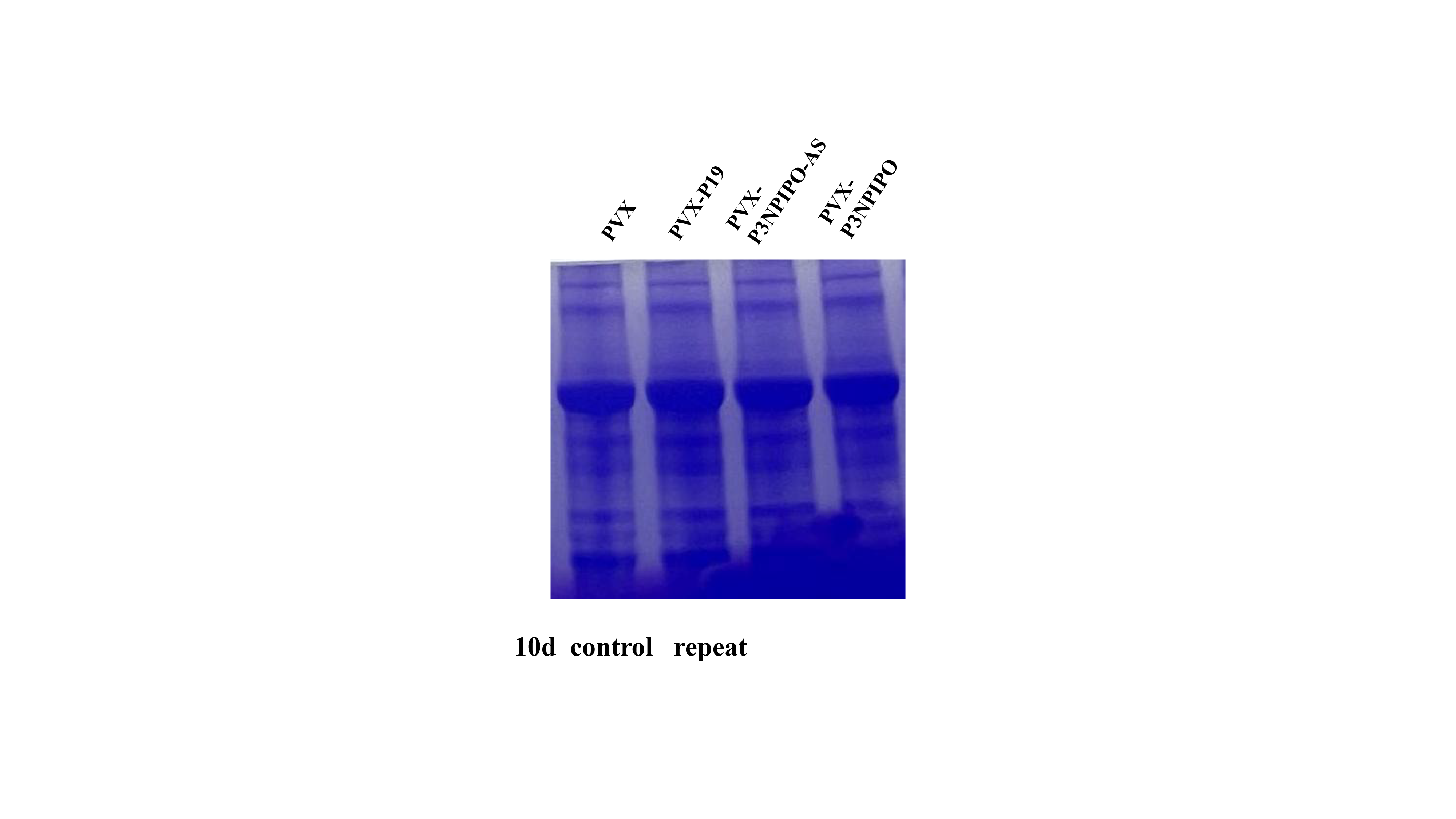

Supplement: Supplementary file 1 [file viruses-14-02171-s001.zip › Figs-original/Fig1-WB-original/Fig.1-10d-control3.tif]

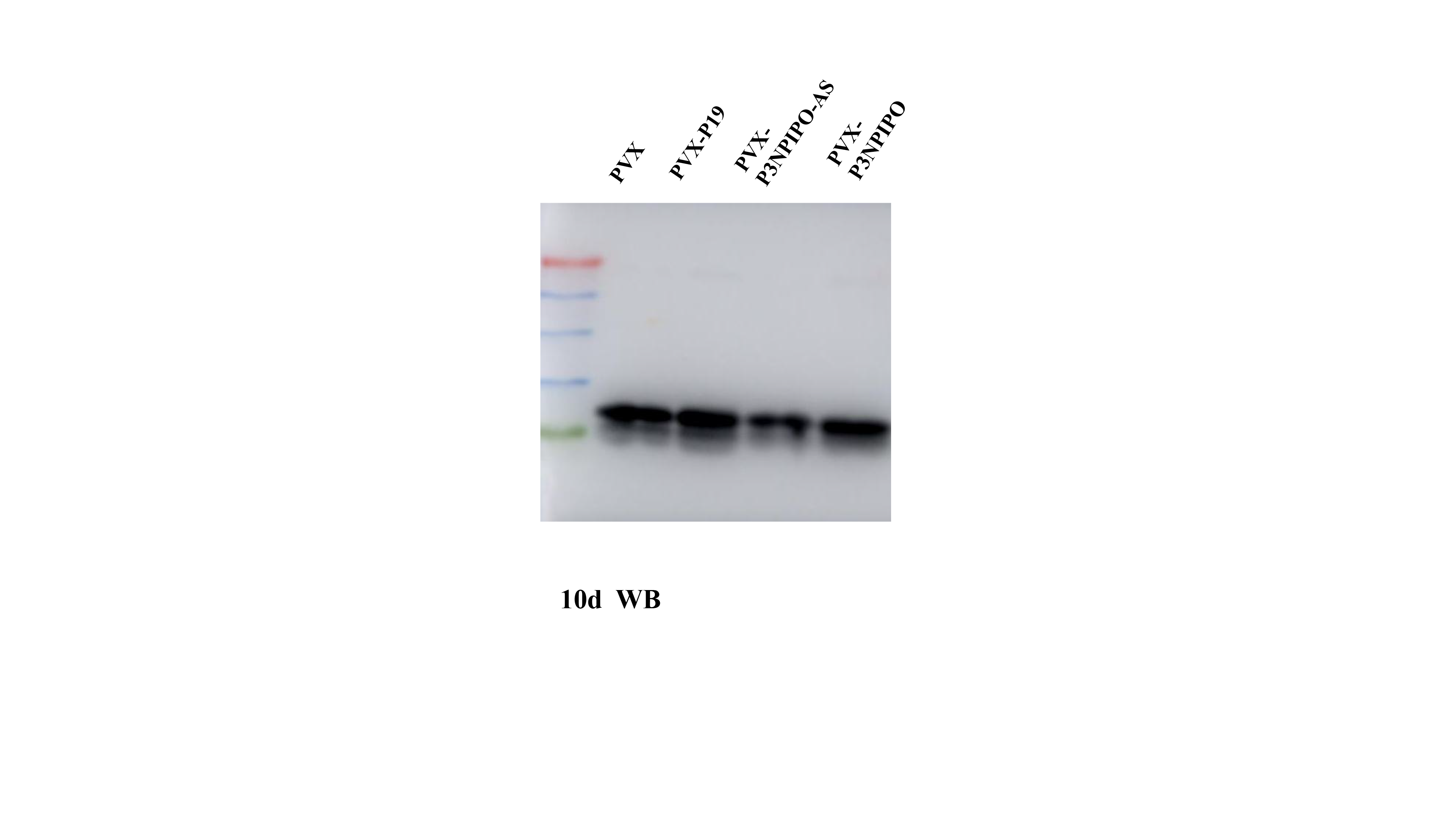

Supplement: Supplementary file 1 [file viruses-14-02171-s001.zip › Figs-original/Fig1-WB-original/Fig.1-10d-WB1.tif]

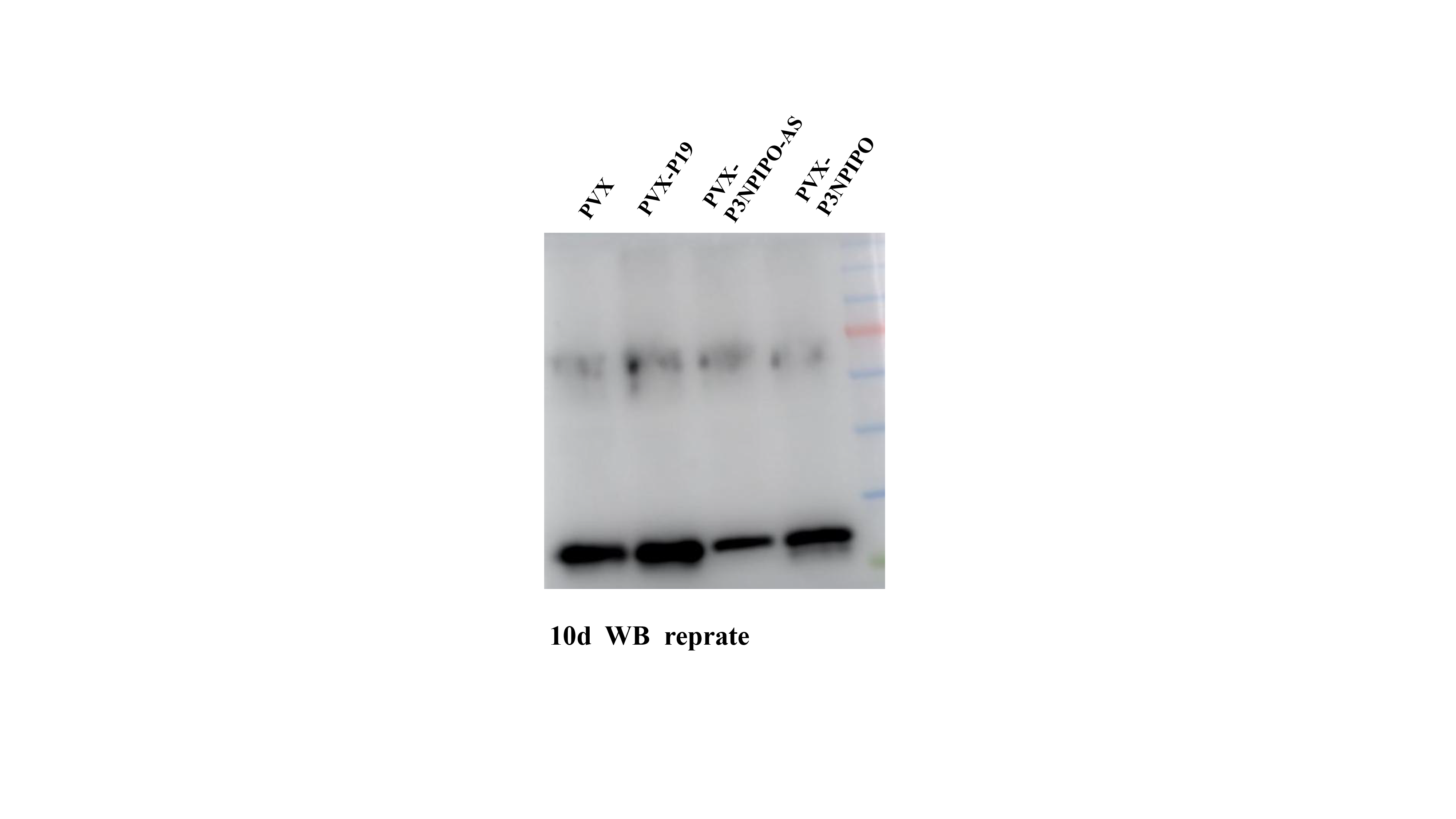

Supplement: Supplementary file 1 [file viruses-14-02171-s001.zip › Figs-original/Fig1-WB-original/Fig.1-10d-WB2.tif]

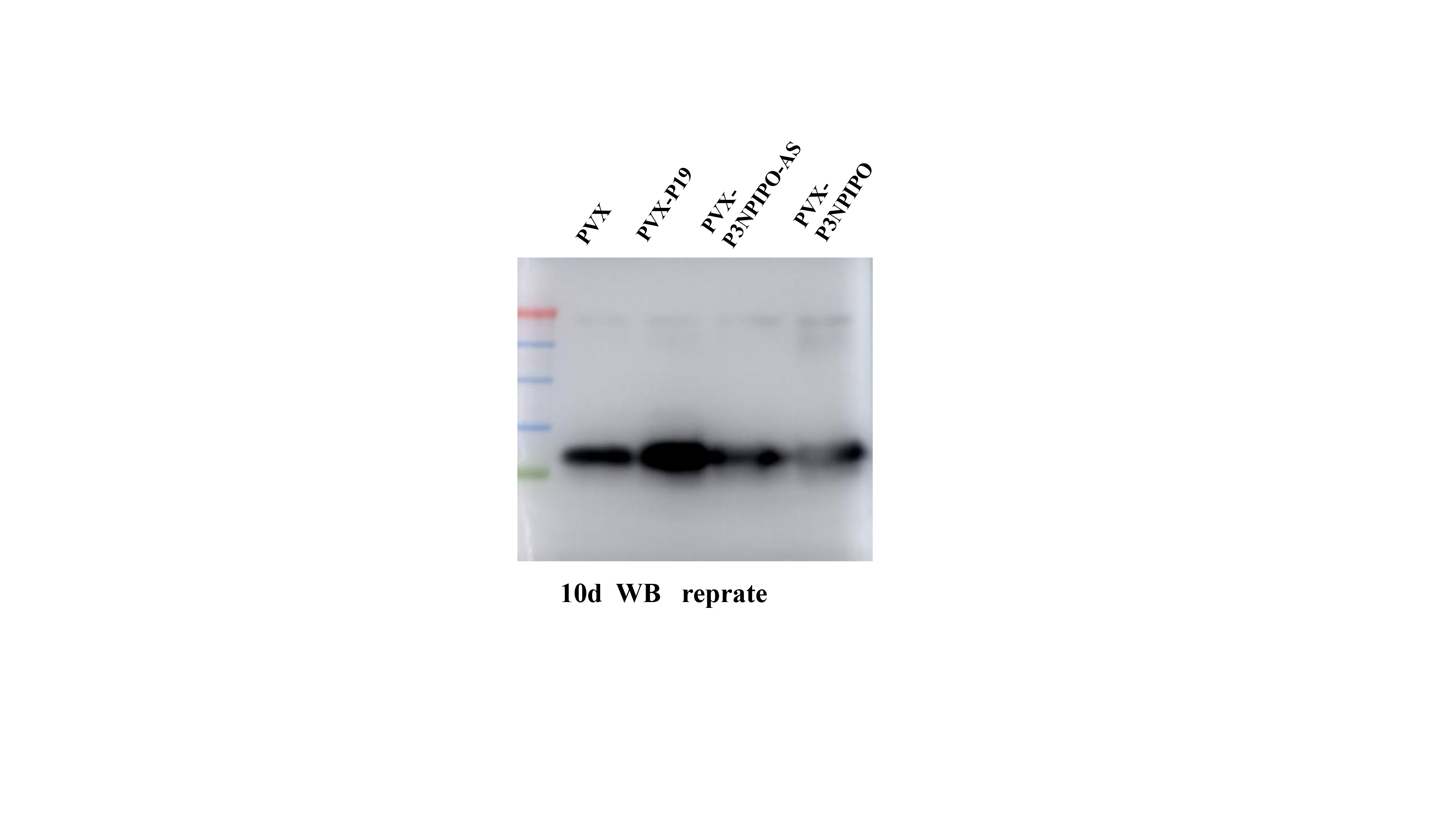

Supplement: Supplementary file 1 [file viruses-14-02171-s001.zip › Figs-original/Fig1-WB-original/Fig.1-10d-WB3.tif]

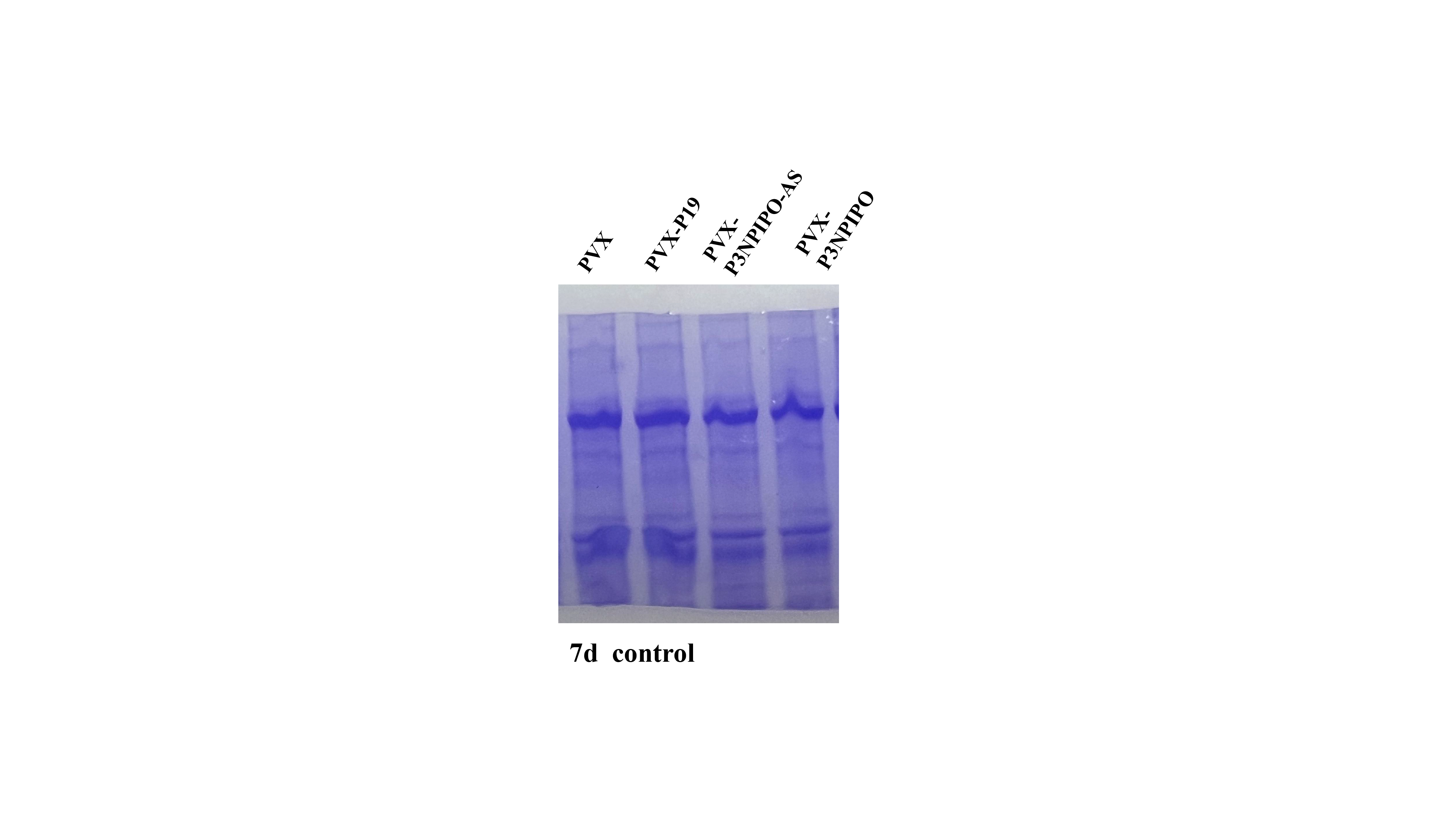

Supplement: Supplementary file 1 [file viruses-14-02171-s001.zip › Figs-original/Fig1-WB-original/Fig.1-7d-control1.tif]

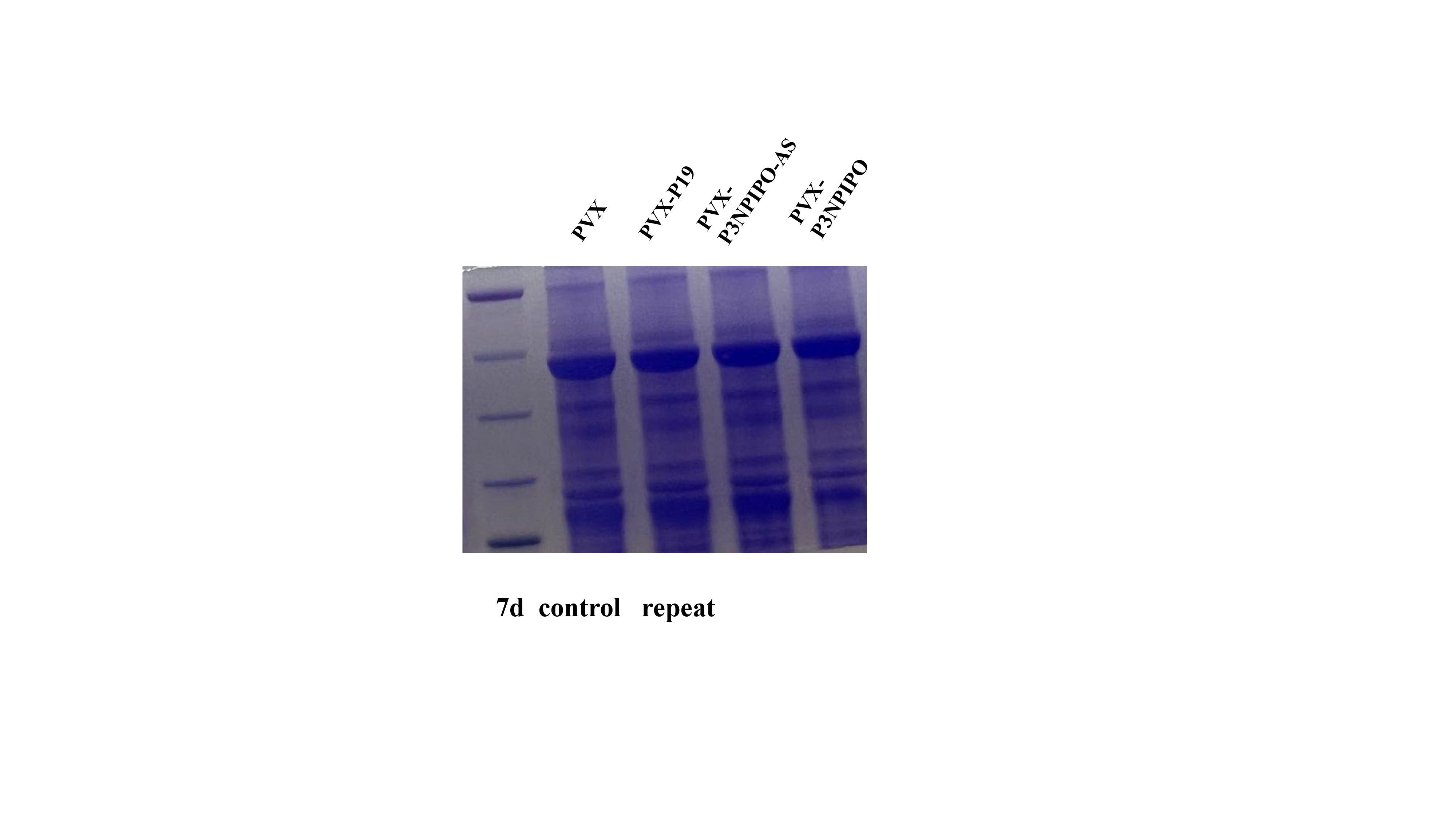

Supplement: Supplementary file 1 [file viruses-14-02171-s001.zip › Figs-original/Fig1-WB-original/Fig.1-7d-control2.tif]

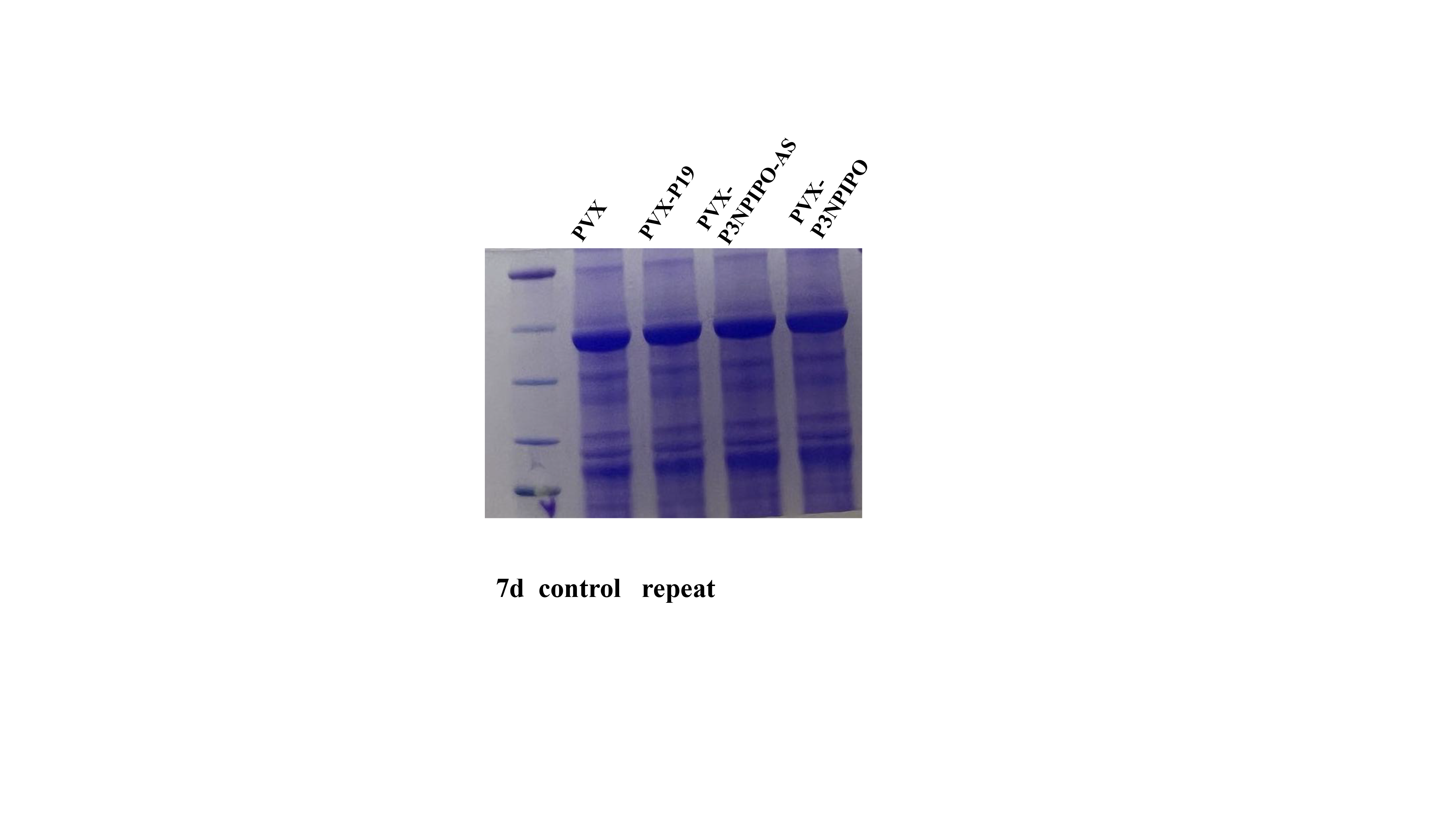

Supplement: Supplementary file 1 [file viruses-14-02171-s001.zip › Figs-original/Fig1-WB-original/Fig.1-7d-control3.tif]

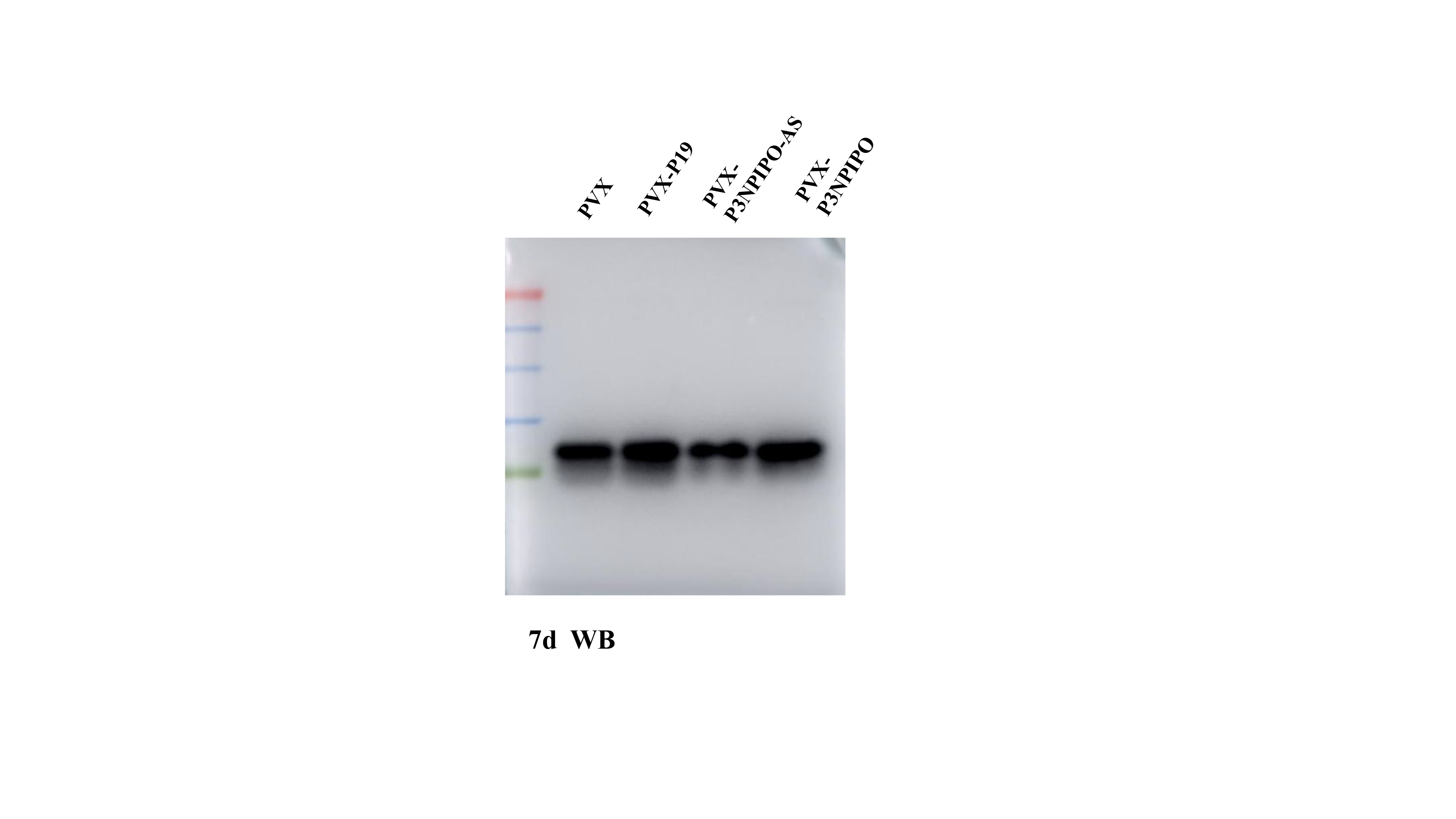

Supplement: Supplementary file 1 [file viruses-14-02171-s001.zip › Figs-original/Fig1-WB-original/Fig.1-7d-WB1.tif]

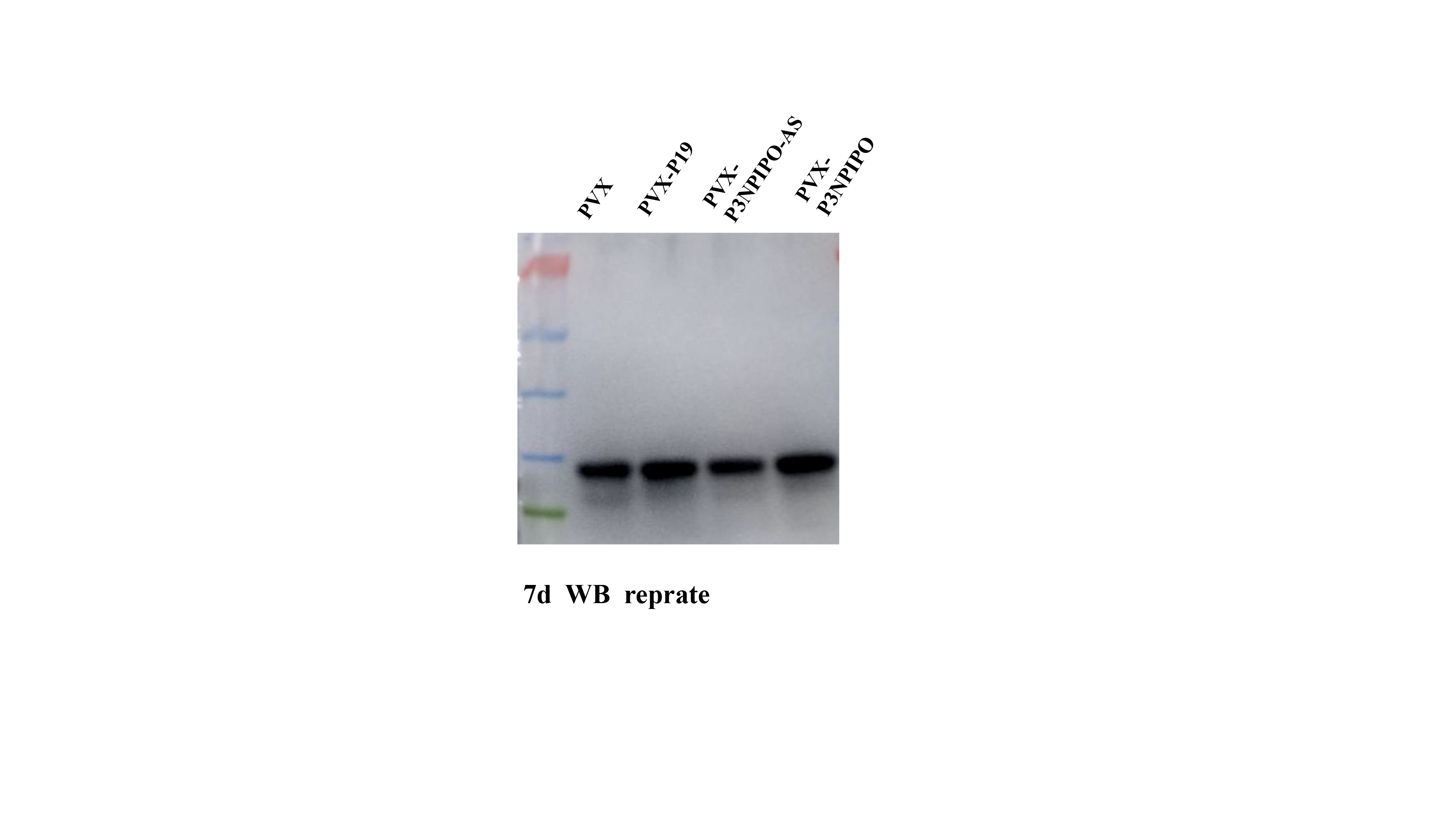

Supplement: Supplementary file 1 [file viruses-14-02171-s001.zip › Figs-original/Fig1-WB-original/Fig.1-7d-WB2.tif]

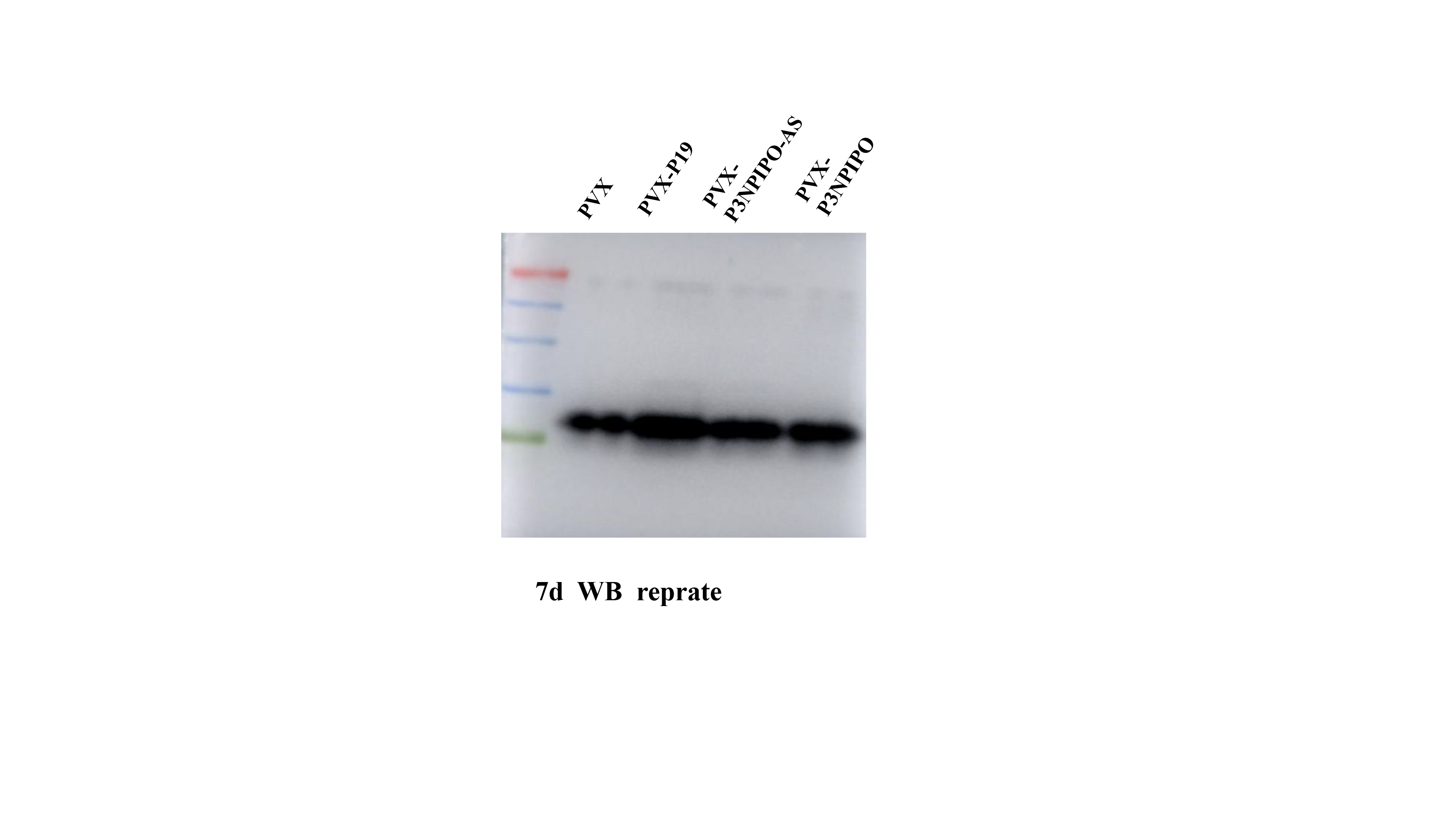

Supplement: Supplementary file 1 [file viruses-14-02171-s001.zip › Figs-original/Fig1-WB-original/Fig.1-7d-WB3.tif]

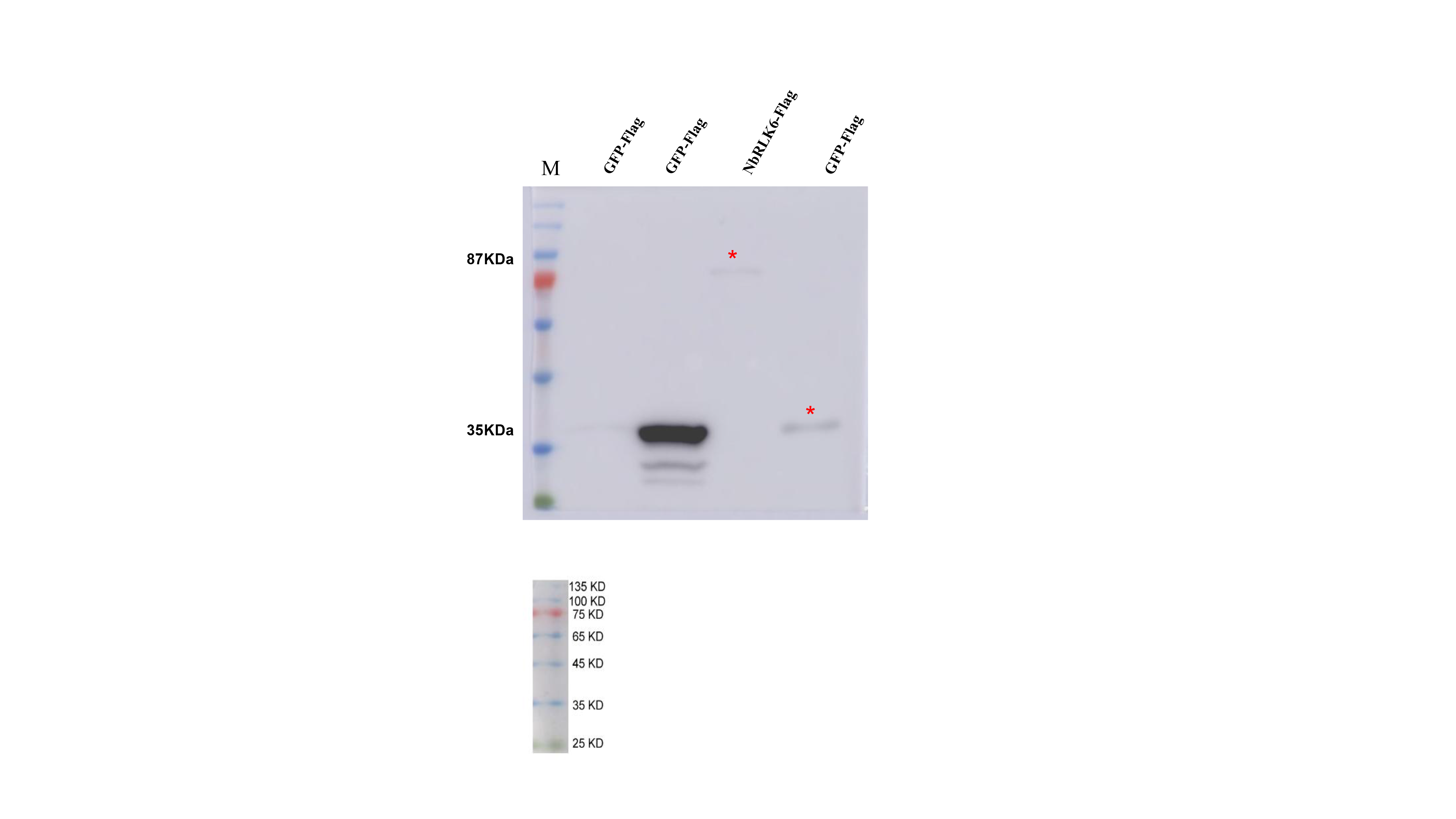

Supplement: Supplementary file 1 [file viruses-14-02171-s001.zip › Figs-original/Fig3 IP-original/input-flag.tif]

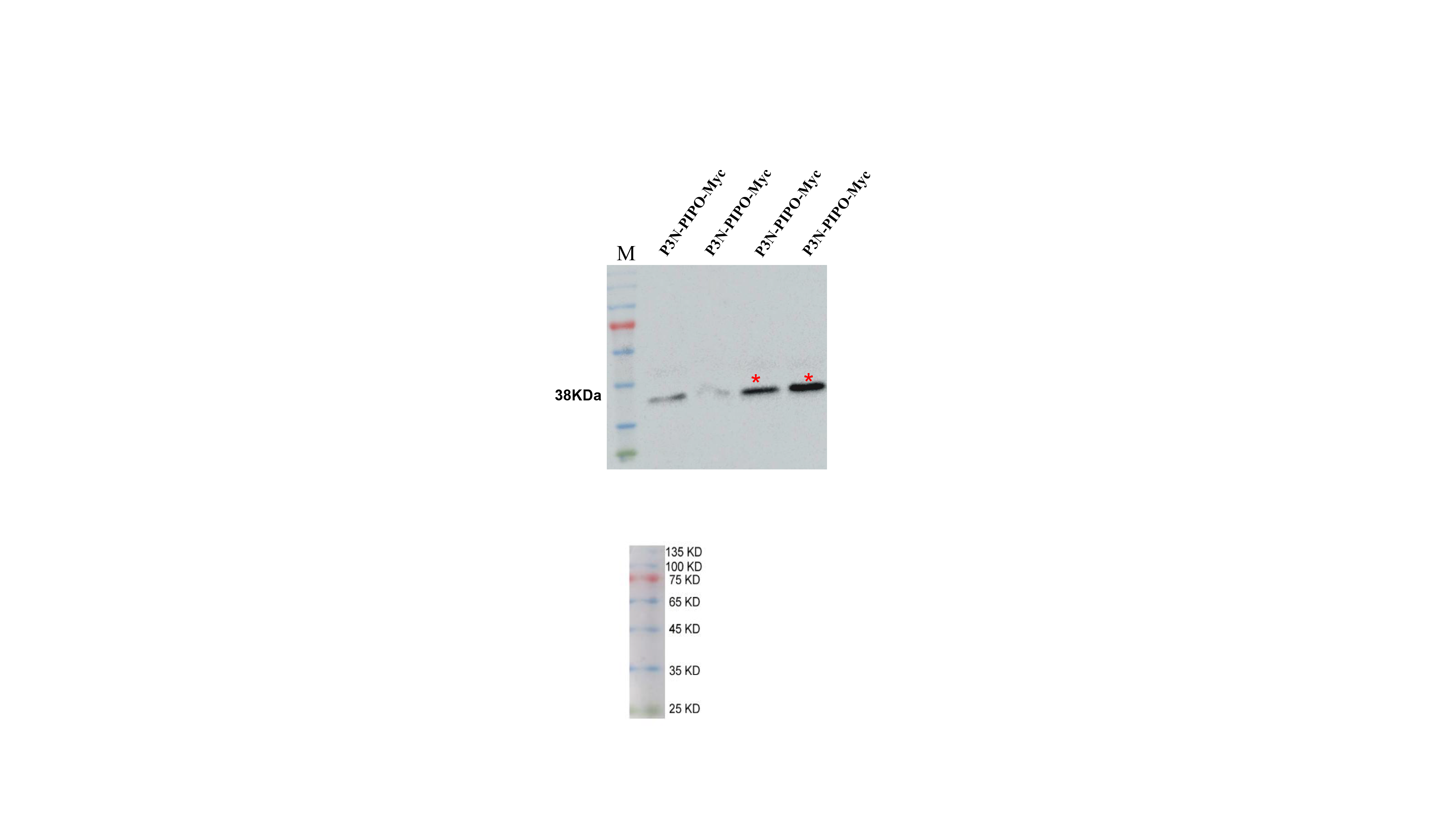

Supplement: Supplementary file 1 [file viruses-14-02171-s001.zip › Figs-original/Fig3 IP-original/INPUT-MYC.tif]

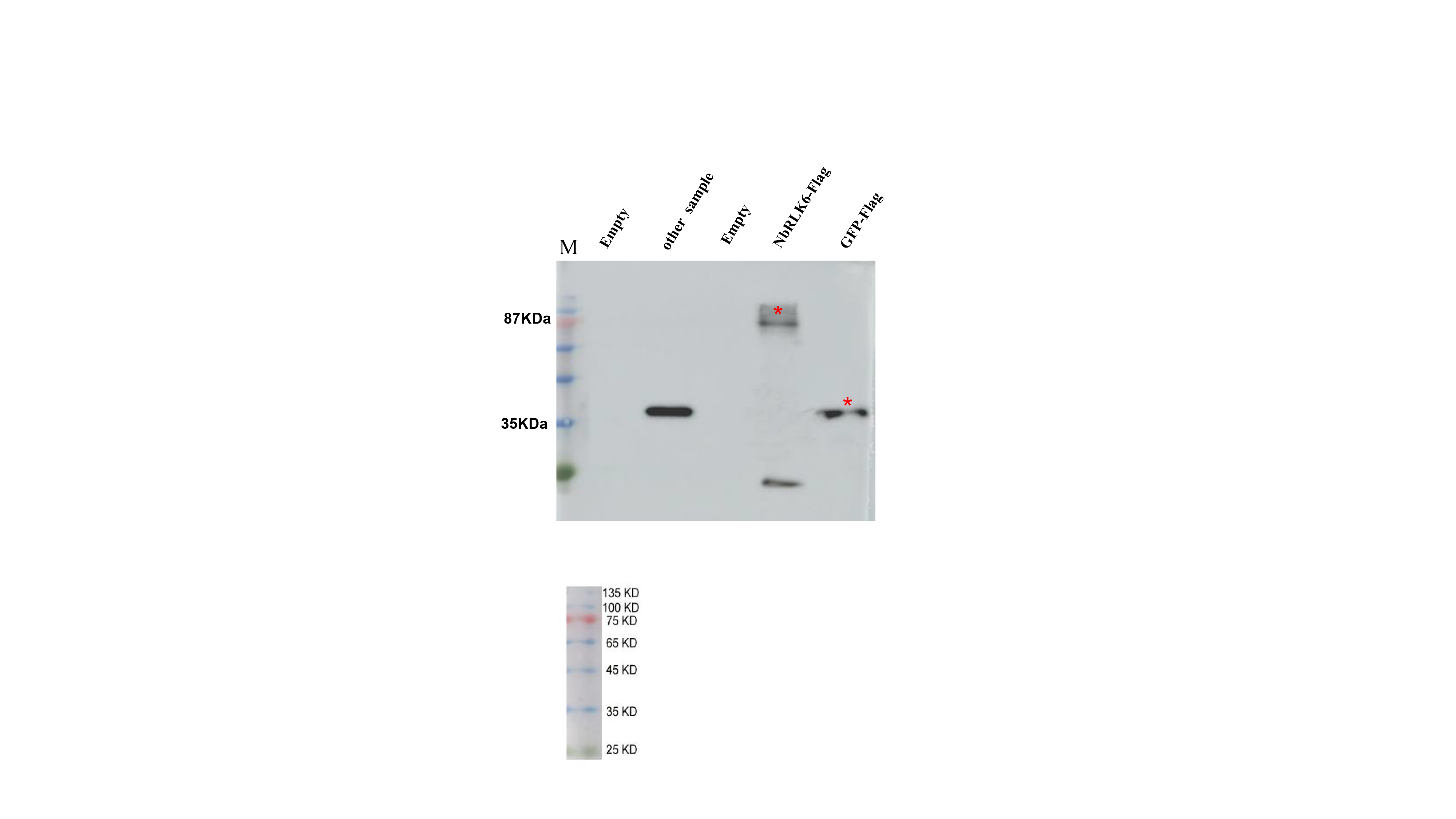

Supplement: Supplementary file 1 [file viruses-14-02171-s001.zip › Figs-original/Fig3 IP-original/IP-Flag.tif]

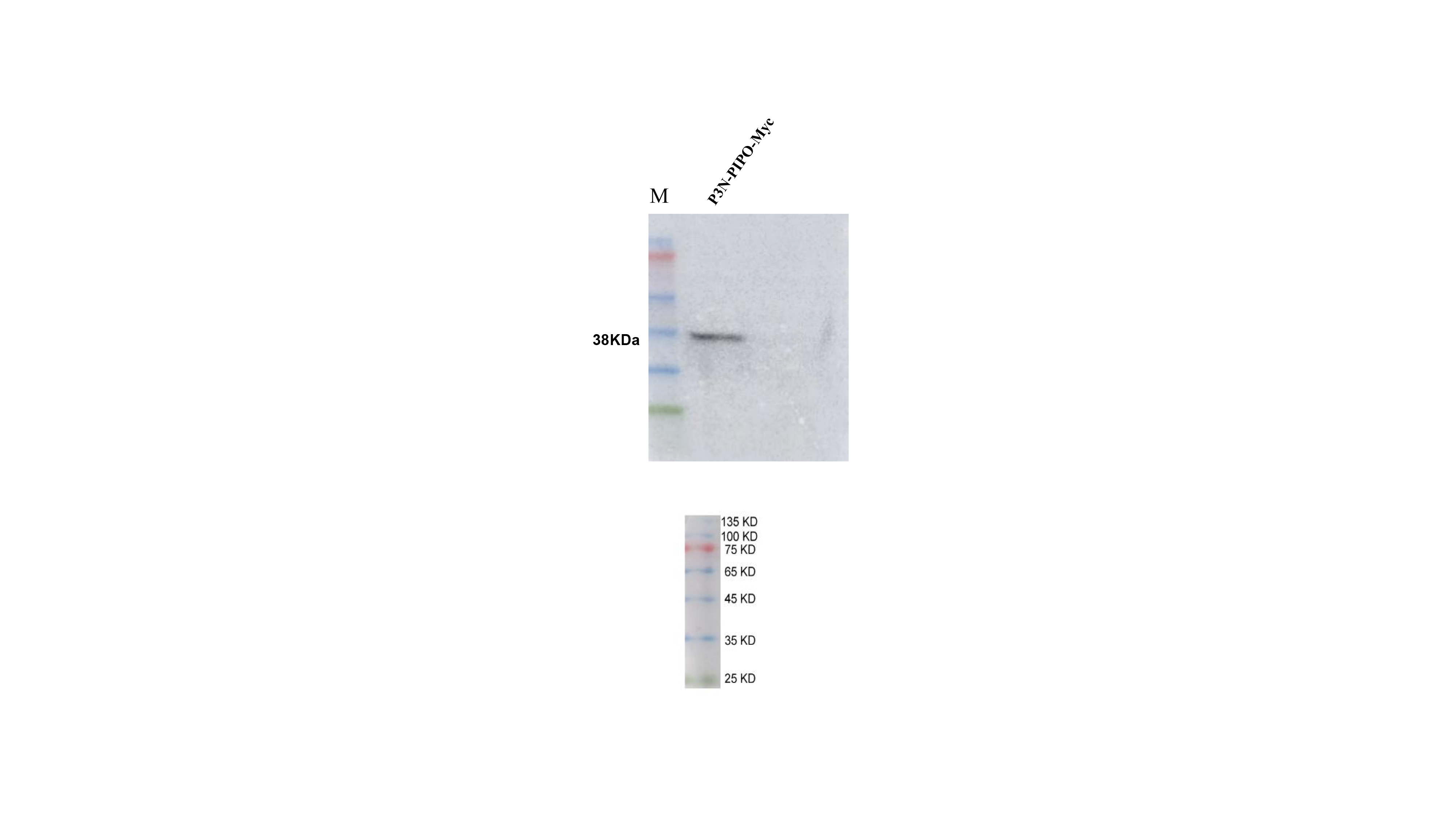

Supplement: Supplementary file 1 [file viruses-14-02171-s001.zip › Figs-original/Fig3 IP-original/IP-Myc.tif]

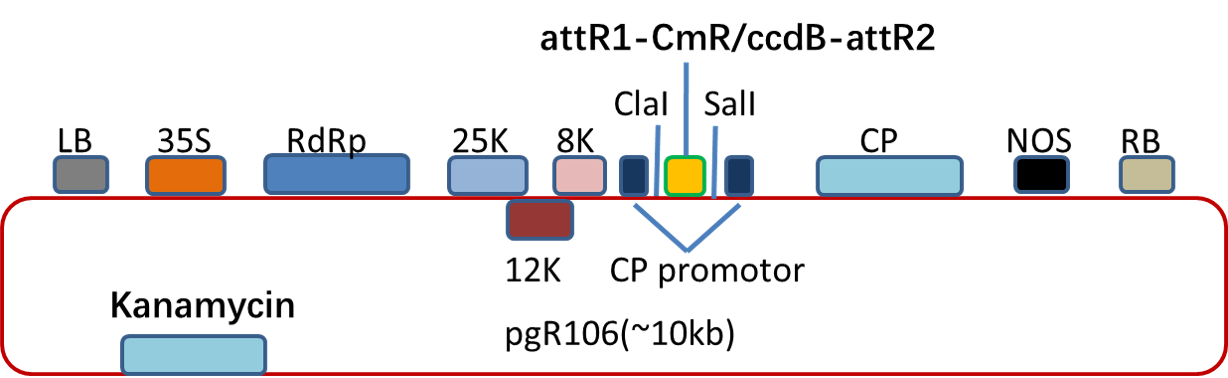

Supplement: Supplementary file 1 [file viruses-14-02171-s001.zip › Figure S1.tif]

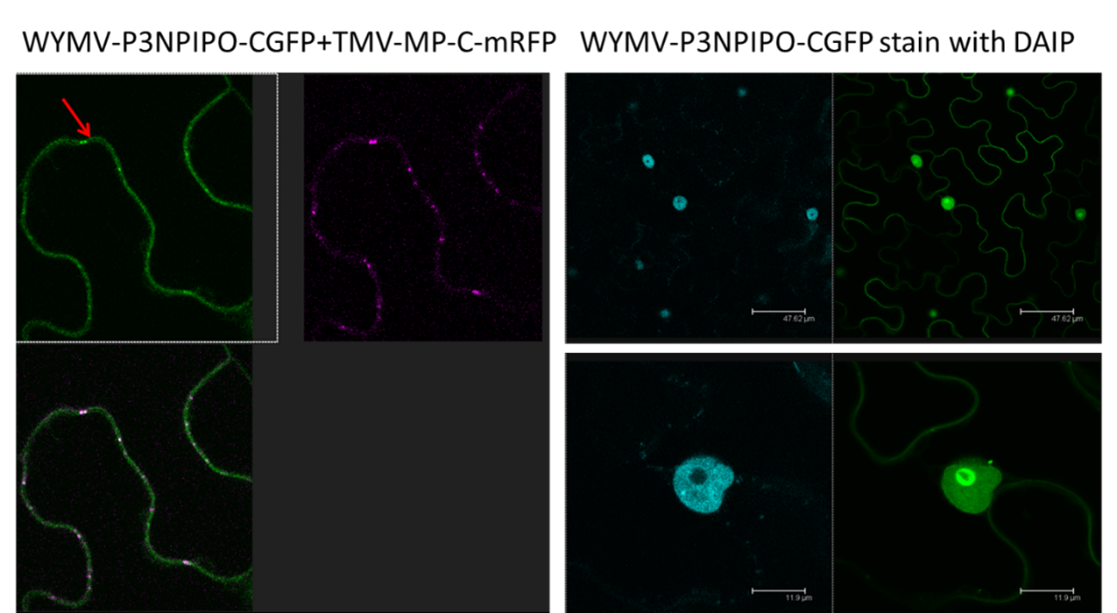

Supplement: Supplementary file 1 [file viruses-14-02171-s001.zip › FigureS2.tif]
